# Acorn Crop, Seed Size and Chemical Defenses Determine the Performance of Specialized Insect Predators and Reproductive Output in a Mediterranean Oak

**DOI:** 10.3390/insects12080721

**Published:** 2021-08-12

**Authors:** Eduardo T. Mezquida, Paula Caputo, Pablo Acebes

**Affiliations:** 1 Department of Ecology, Faculty of Sciences, Autonomous University of Madrid, 28049 Madrid, Spain; paula.caputo93@gmail.com (P.C.); pablo.acebes@uam.es (P.A.); 2 Biodiversity and Global Change Research Center (CIBC-UAM), Autonomous University of Madrid, 28049 Madrid, Spain

**Keywords:** acorn traits, crop size, *Curculio*, *Cydia*, competition, plant–insect interactions, pre-dispersal seed predation, *Quercus faginaea*

## Abstract

**Simple Summary:**

Seed predation is an antagonistic interaction that can limit plant population dynamics. We investigated the interaction between *Quercus faginea* and two specialized pre-dispersal insect seed predators (weevils and moths) during two years of contrasting crop size to determine the consequences of oak reproductive investment on seed production and insect performance. Crop and acorn size were lower during the second year, although seed predation rates by insects were similar during both crop years. Oaks producing more acorns reduced seed predation by insects during the large crop year and thus improved their reproductive success, and those producing bigger acorns experienced higher levels of seed predation, and more insect larvae developed inside the available acorns during the low crop year. Inter- and intra-specific insect competition increased during the low crop year and were affected by tannin content in acorns. Despite substantial between-year variations in crop and acorn size, insect performance was similar due to larvae being able to finish their development by depleting acorn reserves when resources were low. Crop size, acorn size and chemical composition seem important traits for reducing seed predation by specialized insects and improve reproductive success in this Mediterranean oak species.

**Abstract:**

Seed predation is an antagonistic interaction that negatively affects the performance of individual plants and can limit plant population dynamics. In animal-dispersed plants, crop size is an important determinant of plant reproductive success through its effect on seed dispersers and predators. Seed traits, such as size or chemical composition, can also increase the tolerance to seed predators or reduce their performance. We investigated the interaction between *Quercus faginea* and two specialized pre-dispersal insect seed predators (weevils and moths) during two years of contrasting crop size to determine the consequences of oak reproductive investment on seed production and insect performance. Crop size was 44% lower and acorns were 32% smaller in the second year, although acorn predation by insects was proportionally similar between both years at the population level. Individual trees producing larger crops showed a lower incidence of insect predators during the year of abundant acorn production, whereas trees producing bigger acorns experienced higher seed predation rates by insects, and acorns held more insect larvae in the low crop year. Competition between insects increased when acorn production was low, and higher tannin content in acorns further constrained the number of weevil larvae developing together in the same acorn. However, the abundance and size of insect larvae produced per tree were similar between the two crop years, and this was due to larvae often depleting acorn reserves when resources were low. Oak reproductive output increased nearly two-fold during the large crop year. Crop size variation, acorn production in a given year and acorn size and chemical composition seem to be important traits for reducing damage by insect predators in *Quercus faginea* and improve oak reproductive success.

## 1. Introduction

Seed predation is an antagonistic interaction in which animals feed on plant seeds, thus reducing the number of viable seeds produced by plants [1,2]. Seed predators can sometimes kill most of the developing seeds at a local scale or entire crops of individual plants [3,4,5]. However, this initial reduction in the seed crop might not be relevant for plant populations if seeds not consumed by seed predators will die by competition or density-dependent processes at later stages of plant development [4,6]. In that case, seed production by plants would be more determinant for the dynamics of seed predator populations than the impact that seed predators exert on plant populations [7,8]. Conversely, seed predators can negatively affect plant population dynamics by reducing recruitment and lowering population growth [9,10,11,12]. In animal-dispersed plants, the number of seeds produced usually improves plant reproductive success because large crops attract seed dispersers [13,14]. However, more productive plants may also attract seed predators and display higher levels of seed predation [15,16]. The net effect of crop size on plant reproduction will depend on the spatial and temporal variations in seed production among individual plants within the population and the dispersal capacity of the predators [17,18,19].

Oaks (*Quercus* spp.) are nut-bearing plants that produce large nutritive seeds (i.e., acorns) containing the resources for seed germination and early seedling growth [20]. The production of these nutritious food sources attracts animals that feed on the acorns, some of which act as seed dispersers [20,21]. During acorn development, specialized insect predators lay eggs, and their larvae consume the acorn, feeding on the cotyledons and sometimes the embryo [20,22,23]. These pre-dispersal insect predators reduce plant reproductive capacity by decreasing the initial acorn crop for dispersal [24,25]. Oak species produce variable seed crops from year to year [26,27,28]. Years of large synchronized seed crops can reduce the incidence of pre-dispersal acorn predators, and thus their negative cost to plants [3,28,29]. Moreover, low seed crops can cause the poor reproductive performance of seed predators [28,30]. However, insect larvae do not always consume acorns entirely, leaving part of the cotyledons and embryo unharmed [23,31,32]. The extent of acorn consumption by insects depends on acorn size and the number and size of larvae inside the acorn [33,34]. Therefore, when fewer acorns are produced, acorn size will be an important determinant of insect reproductive performance [35,36], and, in turn, on the abundance of insect predators in the next seeding seasons (i.e., the numerical response of the predators; [30,37]).

Here, we focus on the interaction between a Mediterranean deciduous oak, the Valencian oak (*Quercus faginea* Lam.), and two specialized pre-dispersal insect seed predators during two years of contrasting crop size to determine the consequences of oak reproductive investment on seed production and insect performance. Valencian oaks produce variable acorn crops from year to year, with years of exceptional crops followed by several years of medium or low crops, as in other Mediterranean oaks [28,38,39]. As in other oak species, acorns are attacked by acorn weevils (*Curculio* spp., Curculionidae) and moths (*Cydia* spp., Tortricidae), whose larvae develop inside acorns, feeding on the cotyledons, thus reducing the availability of sound acorns for dispersal [40]. First, we quantified crop size, acorn size and acorn predation rates by insects during two consecutive years and address the hypothesis that trees producing greater crops during a year of high acorn production would reduce predation rates by specialized and low mobile insects [31]. During years of lower crops, in turn, we expected that female insects would prefer to oviposit in trees with more and bigger acorns because more females will oviposit in the few acorns available, and acorn size determines the amount of food available for larval development [35,41]. Therefore, we also expected that the number of larvae developing inside acorns would be higher during small crops and that the size of full-grown larvae would depend on acorn size and the number of larvae in the acorn [35,36]. To test this, we examined the number of larvae of each insect per acorn and larval size and their relationship to acorn size and the presence of the larvae of the two insects during two contrasting crop years.

Second, we use the analyses on seed predation rates, the number of larvae per acorn and larval size for each insect during the two crop years to evaluate the interference between the two acorn insects [42], and expected that competition would increase during the year of low acorn production [18]. Third, we examined whether tannin content in acorns deters seed predation by insects. Tannins are a group of phenolic compounds that presumably reduce predation by insect and vertebrate seed predators [20,43,44]. Oak species that produce acorns with high tannin concentrations usually show lower levels of seed predation by acorn weevils [20,45,46], or weevils have lower larval performance [40]. However, the effect of tannin content on acorn predation by insects and insect performance among individual trees has rarely been addressed [45]. Finally, we estimated the number of sound acorns surviving the pre-dispersal period and the number and size of larvae emerging from acorns for each insect species to assess the consequences of distinct annual reproductive investment on seed output and insect performance at individual trees.

## 2. Materials and Methods

### 2.1. Study Area and Organisms

The study area was located in Aranzueque (40°29′ N, 3°4′ W), Guadalajara province, central Spain. The climate is continental Mediterranean with an average annual precipitation of 476 mm and a mean annual temperature between 12–15 °C. The area is flat at elevations about 900 m.a.s.l. and crossed by streams and rivers, with relatively steep slopes between the valleys (around 700 m.a.s.l.) and the upper plateaus. The upper flat areas are dominated by extensive cereal crops and holm oaks (*Quercus ilex* L.) in the non-cultivated parts. The valley and lower slopes are used for cereal and other herbaceous crops. Valencian oaks occupy the slopes with deeper soils, whereas kermes oaks (*Q. coccifera* L.) occur in steep dry slopes with poor soils.

The Valencian oak is a medium-sized deciduous oak tree native to the western Mediterranean region and widely distributed in the Iberian Peninsula. It is a shade-tolerant oak that can grow in a wide range of substrates, topographic locations and climatic conditions, although it prefers base-rich soils [47]. Valencian oaks propagate by seeds and sprout from trunks and roots. Acorns ripen during late September and October, which can potentially be depredated by several weevil species (*Curculio elephas*, *C. glandium* and *C. pellitus*; [40]) and two moth species (likely *Cydia fagiglandana*, although *C. splendana* could also be present [48,49]). Weevil adults collected in the area and emerging from larvae suggest that the main species in our study site was *C. glandium*, although some *C. elephas* individuals were also found. Most moth larvae observed during acorn processing corresponded to *C. fagiglandana*. During summer, when acorns start to grow and have attained a minimum size, female weevils drill a small hole with their elongated snouts to deposit eggs inside acorns [33]. They usually lay one egg per acorn, although several females can lay eggs in the same acorn, so several larvae can develop inside an acorn [50]. Larvae grow, feeding on the cotyledons until completing their development. The number of seed reserves consumed, and therefore the likelihood of consuming and killing the embryo, depend on acorn size and the number and size of larvae growing in the acorn [23,31,49]. The full-grown larvae bore a hole to exit the acorn and bury to overwinter underground. Acorn moths have similar life cycles. Females of *Cydia* moths lay eggs during summer, depositing their eggs on or near acorns. First-instar larvae hatch from eggs and penetrate the acorn, where the larva develop, feeding on seed reserves. After several instars, the larvae leave the acorn to build a silk cocoon in the leaf litter to overwinter [42,48]. Acorn weevils and moths overlap during the acorn infestation period, although weevil larvae hatch inside the acorn and moth larvae hatch outside the acorn, and the larvae enter the acorn. Moth larvae leave a chemical marker when entering the acorn that is detected by female weevils and seems to inhibit weevil egg-laying [42]. Therefore, the presence of larvae of both insect species in the same acorn is likely due to a moth larvae entering an acorn with weevil larvae already present [42]. Acorns fall to the ground near the mother plant and can be dispersed short distances by rodents [51,52] or long distances by birds [53,54].

### 2.2. Sampling Design

During late August 2014, we selected three nearby sites (maximum distance was 4.6 km) and tagged 59 mature oaks with non-overlapping canopies chosen arbitrarily in the three sites. We estimated acorn production of each tree during late September by counting the number of acorns in the canopy within 1 min, 30 s for each half of the crown [55]. In 2015, some of the tagged oaks produced few acorns, so we selected 12 new oaks with acorns near each previously tagged oak and used 39 trees from the previous year for a total of 51 trees. Acorn crop was estimated for the 51 trees as in 2014.

During mid-October of each year, when acorns fully ripen and before seed fall, we collected up to 40 acorns from around the canopy of each tree. In the laboratory, we measured length and maximum width of each acorn to the nearest 0.01 mm with digital calipers. Each acorn was inspected for the presence of larval exit holes and placed in an open plastic vial at room temperature. Acorns were checked every 1–2 days for larval exit for 2–3 months, and each weevil or moth larva exiting acorns was weighted to the nearest 0.1 mg using a digital scale. Afterward, each acorn was opened, and any remaining live larva was weighted or recorded if it was dead.

We used a sample of 568 sound acorns in 2014 and 747 acorns in 2015 (about 10–15 acorns from each tree) to estimate the relationship between acorn size (length and maximum width) and acorn mass. Acorn length and width were measured with digital calipers, and acorn mass was measured to the nearest 0.1 mg with a digital scale after acorns had been oven-dried for 24 h at 40 °C. We also measured seed mass of each acorn without the hard pericarp. We fitted a linear regression between acorn mass and acorn length and width (using log-transformed variables). The model explained 95% of the variation, and regression parameters were used to estimate acorn mass from acorn dimensions of depredated acorns. Acorn mass and seed mass were highly correlated (*r* = 0.99, *p* < 0.001), so we used acorn mass and excluded seed mass from further analyses.

In 2015, we estimated tannin content in acorns by quantifying total phenolic compound contents. Because acorn crop size was low during that year, few sound acorns were available for tannin analyses. We estimated total phenolic compound contents from 2 to 5 acorns from 34 trees. We homogenized the seeds without the hard pericarp using a kitchen blender and lyophilized the resulting coarse powder. Phenolic compounds were extracted, dissolving 7.5 mg of lyophilized seed in 1.5 mL of MeOH:H_2_O (80:20 *v*/*v*), and total phenolic content was determined colorimetrically using a spectrophotometer following Folin–Ciocalteu assay [56]. Two samples from each acorn extract were prepared, and the two absorbance readings at 760 nm were averaged. Total phenolic content was quantified using a calibration curve of absorbance for a standard solution of Gallic acid and expressed in µg/mg of seed [57].

### 2.3. Numerical Analyses

After processing all acorns, we calculated the number of acorns depredated by weevils, moths and by both insects relative to the number of acorns examined for each tree in each year. For each acorn, we summed the number of emergence holes present when measured (each larva bore its own hole to exit the acorn; e.g., [23]), the number of larvae exiting the acorn and any dead larva remaining in the acorn to estimate the number of weevil or moth larvae per acorn and number of larvae per infested acorn were averaged to obtain an estimation at tree level. These estimations are accurate because cannibalism does not occur between the larvae [31,33,42,50]. The weight of larvae developing in the same acorn was averaged for each insect species, and mean larval weight for all infested acorns was calculated for each tree.

#### 2.3.1. Between-Year Variations

We used general or generalized linear models to test for variations in crop size, acorn mass, predation rates by insects, number of larvae per acorn and larval weight between the two years. Error structures used to fit models varied depending on the distribution of the explanatory variables. Crop size was modeled with a negative binomial error structure; acorn mass, number of larvae and larval weight were fitted using a Gaussian error structure; models for predation rates used a binomial error. We checked for overdispersion in models for predation rates, and modeled overdispersion as observation-level random effects [58]. The included random effect was tree number (i.e., a discrete categorical factor varying from 1 to 110, to increase the spread of the distribution [58]).

#### 2.3.2. Acorn Predation by Insects

To test whether acorn predation rates by weevils and moths were determined by tree crop size, acorn mass and the incidence of the other insect at tree level, we used generalized linear mixed models with a binomial error structure for each insect and year. Predictor variables were standardized to zero mean and unit variance, and the model included the main effects, their two-way interactions and the observation-level random effect after checking for overdispersion, as explained above.

#### 2.3.3. Number, Weight and Mortality of Insect Immatures

We used general linear models to test for the effect of tree crop size, acorn mass and the incidence of the other insect on the number of weevil or moth larvae per acorn at tree level. Models for each insect and year included the main effect of predictors and their two-way interactions. To assess whether the weight of the larvae developing in each acorn was affected by acorn mass and the number of larvae of each insect in the acorn, we used general linear models. Because larvae that did not exit and were inside the acorn when processed could have remained in the acorn longer and attained more weight, we included the date of acorn processing in the model as Julian date (day 1 = 1 October) standardized to units of standard deviation. We included the two-way interactions between predictors and initially included tree as a random effect, although variance of the random effect was low and results were similar to models without random effects, so we used the simpler general linear models. Larvae found dead when dissecting acorns were used to calculate immature mortality for each insect relative to the total number of larvae and acorns and compared using Chi-squared tests.

#### 2.3.4. Acorn Tannin Content and Predation by Insects

In order to test whether acorn tannin content influenced weevil and moth predation rates and number of larvae of each insect developing in acorns, we used generalized mixed models with a binomial error distribution for predation rates and general linear models for number of larvae per acorn. Models included acorn tannin content, crop size, acorn mass, predation rate of the other insect and their two-way interactions as predictors at tree level. Mixed models included an observation-level random effect to model overdispersion in the data.

#### 2.3.5. Seed Output and Insect Productivity

We calculated seed output for each tree and year as crop size × proportion of sound acorns. We tested for between-year differences in seed output using a generalized linear model with a negative binomial error structure after rounding values to the nearest integer. In order to obtain an estimation of productivity for each insect, we calculated two composite variables: number of immatures and immature biomass produced per tree. Number of insect immatures produced was calculated as crop size × proportion of acorns with weevils or moths × mean number of weevil or moth larvae per acorn × larval survivorship. Immature biomass was the result of multiplying the previous calculation by mean larval weight. Between-year variations in productivity were tested using generalized linear models with a negative binomial error or Poisson error (moth biomass model).

Modeling was conducted using base packages in R 3.6.1 (R Development Team, Vienna, Austria). Mixed models were fitted using the lme4 [59] and the lmerTest packages [60]. All models were simplified by sequentially removing non-significant terms.

## 3. Results

### 3.1. Crop Size and Acorn Predation by Insects

Crop size and acorn mass were variables between the two years (Table 1, Table S1). Trees produced more and bigger acorns in 2014 compared to 2015, when crop size was on average 44% lower and acorns were 32% smaller (Table 1). Tree crop size and acorn mass were not correlated in 2014 (*r* = 0.17, *p* = 0.20), although showed a positive correlation in 2015 (*r* = 0.40, *p* = 0.004), suggesting that trees investing more in reproduction produced more and larger acorns. The proportion of acorn crop depredated by insects did not differ between the two years (Table 1). Levels of weevil predation were proportionally similar during both years, whereas predation rates by moths were slightly lower in 2015 (Table 1).

### 3.2. Determinants of Seed Predation by Insects

**Figure 1:**
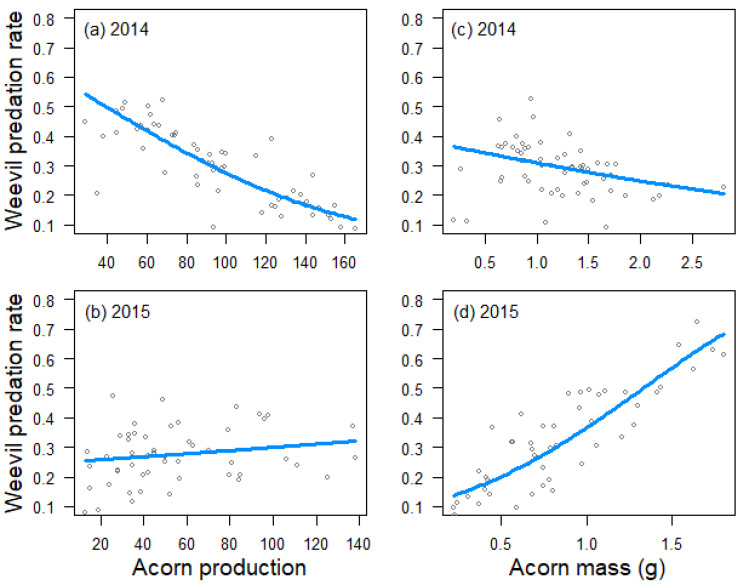
Relationship between tree crop size and weevil predation rate in 2014 (**a**) and 2015 (**b**), and between tree acorn mass and weevil predation rate in 2014 (**c**) and 2015 (**d**), as estimated by generalized linear models. Trend lines fitted to data from 59 *Quercus faginea* trees in 2014 and 51 trees in 2015.

**Figure 2:**
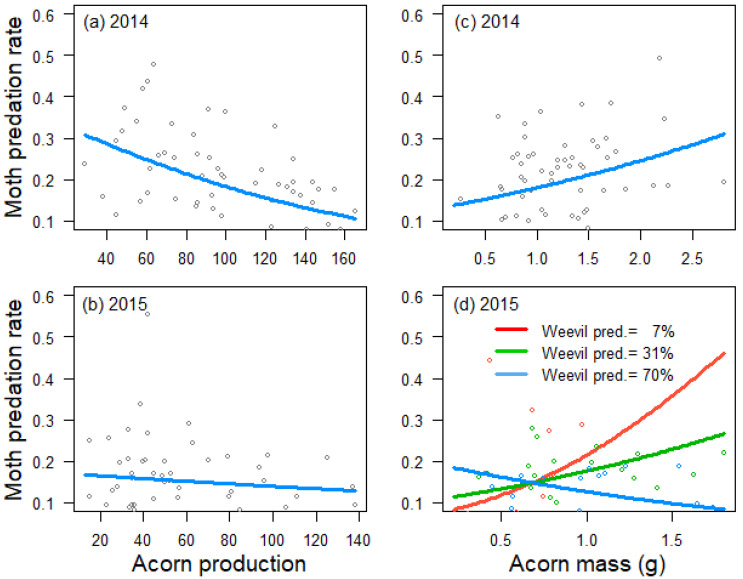
Relationship between tree crop size and moth predation rate in 2014 (**a**) and 2015 (**b**), between acorn mass and moth predation rate in 2014 (**c**) and a cross-sectional plot depicting the interaction between tree acorn mass and weevil predation rate and its effect on moth predation rate in 2015 (**d**), as estimated by generalized linear models. Trend lines fitted to data from 59 *Quercus faginea* trees in 2014 and 51 trees in 2015. Lines in the cross-sectional plot are cross-sections at the tenth (red line), fiftieth (green line) and ninetieth (blue line) percentiles of weevil predation rate in 2015.

Acorn predation by weevils was negatively influenced by tree crop size in 2014 (estimate ± SE: −0.596 ± 0221, *Z* = −2.7, *p* = 0.007; Figure 1a), but not in 2015 (0.082 ± 0.164, *Z* = 0.5, *p* = 0.616; Figure 1b), and positively related to acorn mass in 2015 (0.689 ± 0.152, *Z* = 4.5, *p* < 0.001; Figure 1d), but not in 2014 (−0.157 ± 0.221, *Z* = −0.7, *p* = 0.479; Figure 1c). Crop size also had a negative effect on acorn predation by moths in 2014 (−0.349 ± 0.105, *Z* = −3.3, *p* < 0.001; Figure 2a), but not in 2015 (−0.079 ± 0.130, *Z* = −0.6, *p* = 0.545; Figure 2b). In 2014, moths also preferred trees that produced larger acorns (0.206 ± 0.104, *Z* = 2.0, *p* = 0.047; Figure 2c). In 2015, the best mixed model for moth acorn predation included a negative interaction between acorn mass and the incidence of weevils (−0.311 ± 0.141, *Z* = −2.2, *p* = 0.027), indicating that moths preferred trees producing bigger acorns when the incidence of weevils was low (Figure 2d).

At the tree level, the proportion of acorns infested by both weevils and moths was similar for both years (Table 1), and was positively correlated with predation rates by each insect in 2014 (weevils: *r* = 0.44, *p* < 0.001; moths: *r* = 0.47, *p* < 0.001, *n* = 59 trees), when seed predation by moths was higher; and with predation rates by weevils in 2015 (weevils: *r* = 0.50, *p* < 0.001; moths: *r* = 0.24, *p* = 0.09, *n* = 51 trees), when moth predation was lower. Acorn crop size was not related to infestation rates by both insects (2014: *r* = −0.18, *p* = 0.15; 2015: *r* = 0.04, *p* = 0.78), whereas acorn mass showed a positive correlation in 2015 (*r* = 0.36, *p* = 0.01) and no correlation in 2014 (*r* = −0.01, *p* = 0.93).

### 3.3. Number of Insect Immatures Developing in Acorns

**Figure 3:**
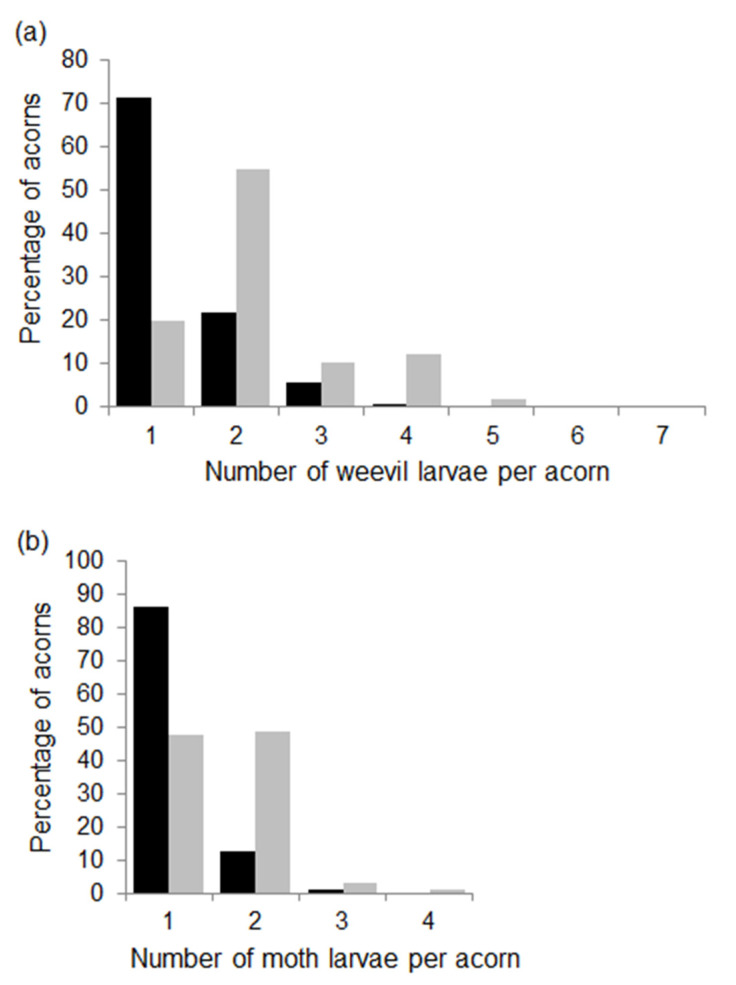
Percentage of acorns with different numbers of weevil (**a**) and moth (**b**) larvae per acorn in the 2014 (black bars) and 2015 (grey bars) fruiting seasons. Percentages were calculated from 619 and 504 acorns infested by weevils and from 345 and 227 acorns infested by moths in 2014 and 2015, respectively.

The number of weevil larvae per acorn ranged from 1 to 6 in 2014, although most larvae (71%) developed without any other weevil larvae (Figure 3a). In 2015, the number of larvae per acorn varied from 1 to 7, and 55% of the acorns had two weevil larvae (Figure 3a). Accordingly, the mean number of weevil larvae per acorn at tree level was higher in 2015 than in 2014 (Table 1). Linear models indicated that the number of weevil larvae increased in trees with larger acorns during both years (estimate ± SE in 2014: 0.253 ± 0.057, *t* = 4.4, *p* < 0.001; 2015: 0.229 ± 0.059, *t* = 3.9, *p* < 0.001). Crop size and the incidence of moths did not correlate with the number of weevil larvae, although in 2015, the model included a negative interaction between acorn mass and predation rates by moths (−0.179 ± 0.079, *t* = −2.3, *p* = 0.029), so greater incidence of moths lowered the slope of the relationship between the number of weevil larvae and acorn mass.

The number of moth larvae per acorn was usually 1 or 2, although the proportion of acorns with one or more larvae per acorn differed between the two years (Figure 3b). In 2014, most acorns infested by moths had one larva (86%), whereas in 2015, the proportion of acorns with one or two moth larvae were similar (Figure 3b). As expected, at the tree level mean, the number of moth larvae per acorn increased from 2014 to 2015 (Table 1). Linear models showed no relationships between the number of moth larvae and tree crop size, acorn mass or the incidence of weevils in either of the two years.

### 3.4. Determinants of Insect Immature Size

The mean larval weight of weevils estimated at tree level did not differ between the two years (Table 1). At the acorn level, in 2014, larval weight was positively correlated with acorn size and negatively with the number of other weevil larvae in the acorn. The Julian date had a negative effect on larval weight, indicating that the larvae weighted and processed later tended to be lighter. There was a significant interaction between acorn mass and the number of weevil larvae in the acorn, showing that the positive relationship between acorn mass and larval weight tends to decrease as the number of larvae increases (Table 2, a). Likewise, in 2015, acorn mass was positively related to larval weight, and the interaction between acorn mass and the larval number was significant, although the main effect of the number of weevil larvae was not significant (Table 2, a). The presence of moth larvae in the acorn had no effect in either year.

Larval weight of moths averaged for each infested tree was similar for both years (Table 1). The larval weight of moths developing in acorns was positively correlated with acorn size and negatively with the number of weevil larva present in the acorn during both years (Table 2, b). In 2015, the Julian date negatively correlated with moth larval weight (Table 2, b).

### 3.5. Insect Larval Mortality

In 2014, mortality of immature weevils was 6.9% (*n* = 845 larvae) that occurred in 8.1% of the acorns (*n* = 619). Larval mortality tended to be lower in 2015, in which 2.1% of the 1125 larvae were found dead in 4.4% of the 504 acorns dissected. Mortality increased with the number of weevil larvae in the acorn in 2014 (3.6%, *n* = 442 acorns, 16.9%, *n* = 136, 26.8%, *n* = 41, for acorns with 1, 2 and 3 or more larvae, respectively; χ^2^_2_ = 45.5, *p* < 0.001). In contrast, larval mortality was similar in acorns with one or more weevil larva in 2015 (5.0%, *n* = 100 acorns, 4.7%, *n* = 227, 3.2%, *n* = 127, for acorns with 1, 2 and 3 or more larvae, respectively; χ^2^_2_ = 0.6, *p* = 0.73).

Larval mortality in moths was 6.8% of the 396 larvae developing in 7.8% of the acorns (*n* = 345) in 2014. In 2015, moth larval mortality was relatively similar (5.3% of the 357 larvae were found dead in 8.4% of the 227 acorns). In each year, larval mortality of moths did not differ in acorns with one or two immature moths (2014, 1 larva: 8.1%, *n* = 298 acorns, 2 larvae: 7.0%, *n* = 43, χ^2^_2_ < 0.001, *p* = 1; 2015, 1 larva: 12.0%, *n* = 108 acorns, 2 larvae: 5.5%, *n* = 110, χ^2^_2_ = 2.2, *p* = 0.14).

### 3.6. Tannin Content in Acorns and Seed Predation by Insects

Acorn tannin content did not correlate with weevil predation rates in 2015, and the main predictor was acorn mass (estimate ± SE: 0.717 ± 0.192, *n* = 34 trees, *Z* = 3.7, *p* < 0.001), as shown above. The mixed model for moths included a positive interaction between acorn mass and tannin content (0.394 ± 0.163, *Z* = 2.4, *p* = 0.016). The mean number of weevil larvae per acorn was positively correlated with tree acorn mass (0.283 ± 0.058, *t* = 4.9, *p* < 0.001) and negatively with tannin content (−0.161 ± 0.058, *t* = −2.8, *p* = 0.009). However, the number of moth larvae per acorn did not show any relationship with crop size, acorn mass, the incidence of weevils or tannin content. Larval mortality was not correlated with acorn tannin content (Spearman rank correlation; weevils: *r_s_* = 0.10, *p* = 0.59, *n* = 34 trees; moths: *r_s_* = −0.05, *p* = 0.78, *n* = 33 trees).

### 3.7. Seed Output and Insect Productivity

Seed output was 1.9 times higher in 2014 (52.6 ± 5.9, *n* = 59 trees) than in 2015 (27.3 ± 2.7, *n* = 51, *Z* = −3.7, *p* < 0.001). The number of weevil larvae produced and their biomass per tree were similar for both years (Table 3). However, moth productivity was lower in 2015, and larval biomass per tree tended to be lower, although not significantly (Table 3).

## 4. Discussion

Our results indicate that individual Valencian oak trees producing larger crops reduced the impact of pre-dispersal insect predators during a year of abundant acorn production. The next year, with lower crops, trees producing bigger acorns experienced higher seed predation rates by insects, and acorns hold more insect larvae (i.e., multi-infestation rate). Despite the increased intra- and interspecific interference between insects in the low crop year, the abundance and size of insect larvae produced per tree were similar between the two crop years. From the plant’s perspective, the output of sound acorns increased nearly two-fold during the large crop year. In addition, tannin content in acorns negatively affected the number of weevil larvae developing together in the same acorn during the year of low acorn production.

Valencian oaks produce variable annual crops [38], although we do not have data on acorn production for other years in our study site. Crop size during the second year was about half of the previous year, but likely, more contrasting crop years will occur in a longer time series, with exceptional mast years and years with no or very few acorns produced [38]. During a year of high seed production, oak trees producing large crops reduced the impact of acorn weevils and moths, similar to findings in other oak species [31,34,41]. Acorns produced by trees in that year were bigger than those produced in the next year. However, acorn size did not influence predation rates by insects during the high crop year, and the size of acorns produced by trees was not related to crop size. Acorn size is an important determinant of the number of weevil larvae developing in the same acorn and the final size attained by weevil and moth larvae [35,36]. Nevertheless, most weevil and moth larvae developed singly in acorns during the high crop year, suggesting that the availability of acorns led to lower competition for oviposition sites [36]. During the low crop year, acorn size was the main determinant of insect seed predation. Acorns produced by trees were smaller than the previous year, presumably as a reproductive adjustment to the available resources [41,61]. Trees that produced bigger acorns were more depredated by insects, and the number of larvae developing in the same acorn was higher due to the low availability of acorns [36]. Most weevil-infested acorns had two larvae growing together, and the frequency of acorns with one or two moth larvae was similar. Conversely, the proportion of acorns infested by both insects was similar in both years, although it was positively correlated with acorn size in the low crop year. Thus, trees producing bigger acorns were more likely infested by weevils and moths and host both insects in the same acorn.

We found evidence of increased interference between weevils and moths when the availability of acorns was low (e.g., [18]). Both insects compete for acorns as a resource for their larvae, although the interaction is indirect because no cannibalism occurs between the larvae [33,35,50]. However, this competition seems to be asymmetric because female weevils avoid laying eggs in acorns with moth larvae inside whereas moth larvae can enter acorns with weevil eggs or larvae present [42]. Moths preferred trees with bigger acorns to lay eggs, although this preference was negatively affected by the incidence of weevils when acorn production was low. Likewise, trees producing bigger acorns had more weevil larvae growing per acorn, yet the incidence of moths had a negative impact on this relationship in the low crop year. Moreover, when larvae of both insects developed in the same acorn, the number of weevil larvae negatively influenced the size attained by moth larvae. Therefore, interspecific interference between insects increased with the incidence of each insect and low availability of resources [18,42,62].

Acorn size was associated with insect performance during both years and with seed predation levels by insects during the low crop year, which is consistent with the influence of acorn size on the amount of energy that the seed contains (i.e., quantity component) [35,40,46]. Moreover, we found that the chemical composition of acorns affected the relationship between the number of weevil larvae and acorn size (i.e., quality component). Tannins are secondary chemical compounds that presumably reduce the digestive efficiency of seed predators because they interfere with protein digestion [20,63]. Indeed, total nitrogen content and tannin content were negatively correlated in Valencian oak acorns (*r* = −0.42, *p* = 0.27, *n* = 28 trees; authors, unpublished data). The tannin content in acorns of different oak species seems to reduce weevil predation rates and weevil performance [40,45]. For example, a study in a Mediterranean mixed-oak forest found significant variations in the larval size of the same weevil species growing in acorns of different oak species after controlling for the amount of cotyledon consumed by the larvae, suggesting that the chemical composition of acorns influenced larval development [40]. Our results indicate that the size and chemical composition of acorns limited weevil reproduction at individual trees [36,46], although this effect could be tested only during the low crop year with increased competition for ovipositing sites.

The number and size of insect larvae produced are important traits that influence the numerical response of insect populations in subsequent seeding seasons [19,30]. For example, larval size in acorn weevils has been associated with several key fitness components in later life stages, such as larval survival during the diapause phase buried in the ground, size of emerging adults and potential female fecundity [33]. We found that the number and size of insect larvae, or combined as insect biomass, produced during the high and low crop years were similar overall. During the low crop year, insects competed for acorns, which were scarcer and smaller than the previous year, so multi-infestation events were more common. To attain their final size under increased competition, larvae were more likely to completely deplete acorn reserves [35,50]. In the low crop year, we recorded whether acorns were completely consumed or estimated the proportion of the cotyledons remaining and detected that 66% of depredated acorns (*n* = 685) were depleted (Figure S1). We did not quantify the amount of cotyledon consumed in the high crop year, although acorn depletion was relatively uncommon. Therefore, insect performance was similar between both years due to increased multi-infestation and cotyledon consumption during the low crop year. We note that here we have focused on the mature acorn crop and did not consider the larvae developing in abscised acorns. Because fewer and smaller acorns were produced in the low crop year, larvae developing in abscised acorns were probably more constrained during that crop year [50]. On the other hand, larval mortality tended to increase in the high crop year despite higher multi-infestation and lower resources for the developing larvae in the low crop year. Larval mortality in weevils is usually low during the development period inside the acorn and did not increase as a result of conspecific competition in a study on the chestnut weevil, *Curculio elephas* [33,50]. The higher larval mortality rates during the high crop year were likely due to the increased incidence of parasitoid wasps [30].

The number of acorns produced by Valencian oaks that survived the pre-dispersal period was about double in the high compared to the low crop year. The greater output of sound acorns usually translated into increased seedling recruitment in other oak species as a result of the greater effectiveness of seed-disperser animals [3,20,64]. During the high crop year, trees producing more acorns were proportionally less infected by insects and thus supplied more sound acorns. In the low crop year, trees producing bigger acorns experienced higher insect predation rates. However, crop size and acorn mass were positively correlated at the tree level, suggesting no trade-off in the allocation of resources to both traits [61]. Therefore, tree crop size was also an important trait, determining the supply of sound acorns in the low crop year (correlation between seed output and crop size: *r* = 0.70, *p* < 0.001, *n* = 51 trees). In addition to the greater output of sound acorns during the high crop year, depredated acorns were often partially consumed by insects during that year, whereas, in the low crop year, most depredated acorns were depleted (see above). Acorns with the cotyledons partially consumed by insects can somewhat germinate and produce viable seedlings [31,36], increasing the potential for recruitment during the high crop year. However, the likelihood of germination and seedling establishment in acorns infested by insects is generally low [31,36,40], and they are more likely rotten by microbes, depredated, not removed or left uncached [20,65,66]. Consequently, trees producing large crops supply more sound acorns and likely increase their reproductive success [64].

In this study, we compared two years with contrasting acorn crops, although more contrasting crop years will likely occur in a longer time series (see above). Therefore, we would like to note that other mechanisms besides crop size might shape inter-annual variations in the abundance of insect seed predators and, consequently, seed predation rates. For example, other potential factors influencing this interaction include crop sizes during previous years [62], survival of buried larvae during the diapause period [67], length of larval diapause period [67,68], environmental conditions affecting adult insect emergence from the soil [69] and the use of alternative hosts by insects with greater flight capacity during years of low acorn production [30].

In summary, the production of large acorn crops by individual Valencian oaks was an effective mechanism, reducing the impact of specialized pre-dispersal insect predators. During a low crop year, insect predators could cope with lower acorn availability by increasing the number of larvae developing together in the same acorn and the consumption of most acorn reserves, resulting in similar insect productivity than that during the high crop year. Therefore, years with even lower crops would be needed to affect insect populations. Crop size did not reduce acorn predation rates during the low crop year, although the production of more acorns led to the supply of more sound acorns, so crop size was also an important trait enhancing tree reproductive output in years of intermediate to low crops. Acorn quality (i.e., size and chemical composition) also affected insect predators during the low crop year by limiting the number of larvae developing inside acorns. Therefore, the number of acorns produced in a given year, crop size variation among years and chemical composition of acorns seem three important traits for reducing damage by pre-dispersal insect predators in the Valencian oak.

## Figures and Tables

**Table 1:** Crop size and acorn mass for Valencian oak trees, and seed predation rates, number of larvae per acorn and larval weight for two pre-dispersal insect predators in two years with contrasting acorn production in a population in central Spain. Values are mean (SE), and *Z* or *t* statistics and associated *p*-values from mixed or linear models comparing between-year differences for each variable. Sample size was 59 and 51 trees in 2014 and 2015, respectively, except for variables on larval weight, in which the number of trees is indicated after their parameters.

Variables	2014	2015	*Z* or *t*	*p*
Acorn production	98.8 (0.6)	55.3 (4.9)	−6.3	<0.001
Acorn mass (g)	1.22 (0.07)	0.84 (0.06)	−4.3	<0.001
Overall acorn predation (%)	51.4 (4.2)	46.8 (3.4)	−1.2	0.231
Acorn predation by weevils (%)	35.3 (3.8)	34.5 (3.3)	−0.2	0.828
Acorn predation by moths (%)	20.6 (1.7)	15.4 (1.6)	−2.0	0.046
Acorn predation by both weevils and moths (%)	4.1 (0.6)	3.1 (0.6)	−1.6	0.115
Number of weevil larvae per acorn	1.3 (0.1)	2.0 (0.1)	5.6	<0.001
Number of moth larvae per acorn	1.0 (0.0)	1.4 (0.1)	3.9	<0.001
Weevil larval weight (g)	0.0463 (0.0029) 49	0.0483 (0.0022) 51	0.5	0.584
Moth larval weight (g)	0.0210 (0.0011) 51	0.0210 (0.0011) 47	−0.003	0.997

**Table 2:** Estimates and SE from general linear models for larval weight of weevils (a) and moths (b) developing in acorns of *Quercus faginea* during the 2014 and 2015 acorn crops.

Variables	2014			2015		
	Estimate (SE)	*t*	*p*	Estimate (SE)	*t*	*p*
(a) Weevil larval weight						
Intercept	0.060 (0.003)	20.4	<0.001	0.056 (0.003)	20.6	<0.001
Acorn mass	0.016 (0.002)	6.7	<0.001	0.023 (0.002)	9.3	<0.001
Number of weevil larvae	−0.010 (0.002)	−5.8	<0.001	<−0.001 (0.001)	−0.1	0.904
Julian date	−0.007 (0.001)	−5.6	<0.001			
Acorn mass x weevil larvae	−0.003 (0.001)	−2.5	0.012	−0.004 (0.001)	−5.0	<0.001
(b) Moth larval weight						
Intercept	0.022 (0.001)	31.8	<0.001	0.022 (0.001)	24.8	<0.001
Acorn mass	0.004 (0.001)	6.1	<0.001	0.004 (0.001)	5.0	<0.001
Number of weevil larvae	−0.004 (0.001)	−3.5	<0.001	−0.004 (0.001)	−3.7	<0.001
Julian date				−0.004 (0.001)	−4.4	<0.001

**Table 3:** Number and biomass (g) of immature weevils and moths produced per *Quercus faginea* tree in two consecutive fruiting seasons. Values are mean ± SE (number of trees), *Z* statistics and associated *p*-values from generalized linear models testing for differences in productivity variables between the two years.

Insect Productivity	2014	2015	*Z*	*p*
Number of immature weevils per tree	40.9 ± 5.2 (59)	47.7 ± 7.7 (51)	0.7	0.477
Weevil biomass per tree	2.00 ± 0.25 (53)	2.76 ± 0.52 (51)	1.4	0.156
Number of immature moths per tree	20.0 ± 1.9 (59)	12.5 ± 1.6 (51)	−2.7	0.006
Moth biomass per tree	0.44 ± 0.05 (55)	0.27 ± 0.04 (51)	−1.9	0.053

## Data Availability

The data presented in this study are available in Supplementary Materials.

## Supplementary Materials

The following are available online at https://www.mdpi.com/article/10.3390/insects12080721/s1, Figure S1: Number of acorns according to the percentage of the cotyledons consumed by two pre-dispersal insect predators developing in acorns of *Quercus faginea* during the 2015 acorn crop. Table S1: Excel file with the data presented in this study.
