# Supplementary material for: Acorn Crop, Seed Size and Chemical Defenses Determine the Performance of Specialized Insect Predators and Reproductive Output in a Mediterranean Oak
Source: Insects. 2021 Aug 12;12(8):721. doi: 10.3390/insects12080721 (PMC8396859; doi:10.3390/insects12080721)
Supplement: Supplementary file 1 [file insects-12-00721-s001.zip › Supplementary Materials/Figure S1.pdf]

Supplementary Materials

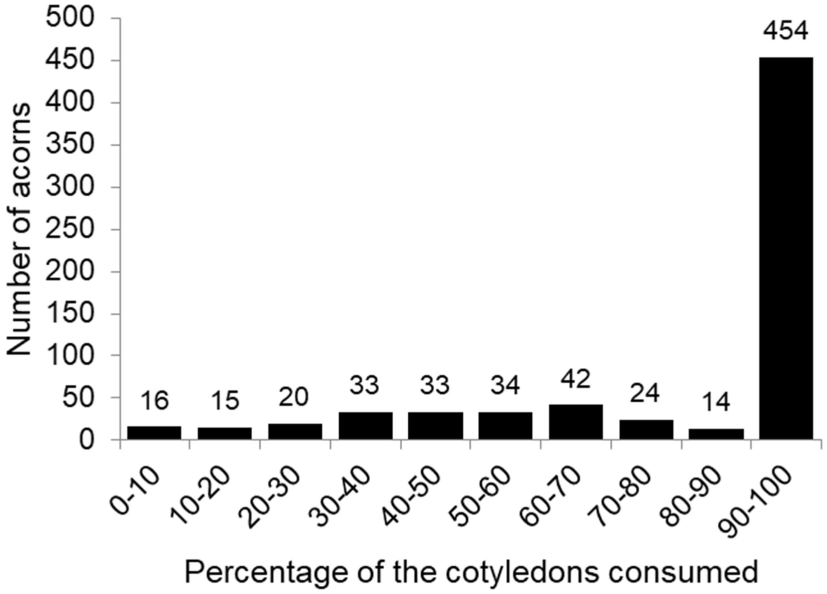

Figure S1. Number of acorns according to the percentage of the cotyledons consumed by two pre-dispersal insect predators developing in acorns of *Quercus faginea* during the 2015 acorn crop.
